# CohortCharacteristics: an R package for population characterisation in observational studies using the OMOP common data model

**DOI:** 10.1007/s10654-025-01352-4

**Published:** 2026-04-03

**Authors:** Mike Du, Albert Prats-Uribe, Núria Mercadé-Besora, Kim Lopez-Guell, Yuchen Guo, Marta Alcalde-Herraiz, Xihang Chen, Antonella Delmestri, Wai Yi Man, Talita Duarte-Salles, Anna Palomar, Agustina Giuliodori, Emanuel Brađašević, Antea Jezidžić, Elvira Bräuner, Susanne Bruun, Katia Verhamme, Mees Mosseveld, James T. Brash, Dina Vojinovic, Isabella Kaczmarczyk, Akram Mendez, Peter Rijnbeek, Daniel Prieto-Alhambra, Edward Burn, Martí Català

**Affiliations:** 1 Pharmaco- and Device Epidemiology Group, Health Data Sciences, Botnar Research Centre, NDORMS, University of Oxford, Windmill Road, Oxford, OX3 7LD UK; 2 Department of Medical Informatics, Erasmus University, Rotterdam, The Netherlands; 3 Data Analytics Center, Danish Medicines Agency, Copenhagen, Denmark; 4 Fundació Institut Universitari per a la recerca a l’Atenció Primària de Salut Jordi Gol i Gurina (IDIAPJGol), Barcelona, Spain; 5 Division for Medical Informatics and Biostatistics, Croatian Institute of Public Health, Zagreb, Croatia; 6 IQVIA, Real World Solutions, London, UK; 7 IQVIA, Real World Solutions, Amsterdam, Netherlands

**Keywords:** Characterisation, Observational studies, Common data model, Epidemiology, R, OMOP CDM

## Abstract

Describing cohort characterisation ensures comparability and reproducibility in multi-database observational studies. To address this need, we developed CohortCharacteristics, an open-source R package that facilitates standardised cohort characterisation in datasets mapped to the Observational Medical Outcomes Partnership (OMOP) Common Data Model (CDM). This study aims to explain the development of the package and demonstrate its core functionality. We developed CohortCharacteristics, an open-source R package that can perform cohort characterisation for various types of databases. To demonstrate its functionality, we then used CohortCharacteristics to generate descriptive statistics on demographics, comorbidities, medication exposures, cohort overlap, and timing of cohort entries. The study included data from CPRD GOLD (UK), DK-DHR (Denmark), IPCI (Netherlands), IQVIA Longitudinal Patient Database Belgium (IQVIA LPD Belgium), IQVIA DA Germany, NAJS (Croatia), and SIDIAP (Spain), all mapped to the OMOP CDM. The CohortCharacteristics R package is freely available on CRAN with detailed vignettes and documentation on its functionality. Cohort characteristics were generally consistent across databases, with similar age distributions and female representation. CPRD GOLD, NAJS, and SIDIAP exhibited higher prescribing rates for respiratory, cardiovascular, and nervous system medications, while IQVIA databases and DK-DHR reported lower rates. Timing analysis showed that dementia diagnoses typically followed insomnia diagnoses in several databases, supporting existing literature. Antipsychotic prescriptions often occurred after dementia diagnosis, reflecting prescribing practices aligned with clinical guidelines. CohortCharacteristics enables consistent cohort characterisation across a network of data mapped to the OMOP CDM, thereby improving transparency in multi-database research. The package’s functionality, demonstrated in this study, illustrates its applicability in observational studies with OMOP CDM data.

**Supplementary Information:**

The online version contains supplementary material available at 10.1007/s10654-025-01352-4.

## Background

A cohort refers to a group of individuals who meet predefined inclusion criteria within a specified time frame. Cohort definitions are fundamental to epidemiological studies conducted with routinely collected healthcare data [1]. Characterising these cohorts provides researchers with essential information about the baseline characteristics of patients within the cohort, such as age, sex, comorbidities and medication exposure histories. This information helps to assess whether the cohort represents the population of interest for the study [2], or to identify differences in baseline characteristics between individuals receiving different interventions in a characterisation study. Additionally, it helps to identify potential heterogeneity related to the data sources and countries specific differences. It also supports comparability of results across different studies, which are key to generalising findings and ensuring reproducibility of results.

The Observational Medical Outcomes Partnership (OMOP) Common Data Model (CDM) provides a standardised structure and vocabulary for capturing healthcare data [3]. Adopting the OMOP CDM allows the use of the same analytic code against multiple data sources, enabling distributed network studies. In network studies, each site executes a shared analytic code locally and returns aggregated results without sharing patient-level data. Developing robust, open-source pipelines can help facilitate such research, reduce the time required to perform it and increase the trust in the results generated. In particular, R is a widely used programming language in OMOP CDM related research.

The OMOP CDM is maintained and supported by the Observational Health Data Sciences and Informatics (OHDSI) initiative, a global open-science community focused on generating reliable real-world evidence [4]. OHDSI also provides a comprehensive ecosystem of open-source tools built around the OMOP CDM, including ATLAS for cohort definition and characterisation [5], and a suite of other R packages collectively known as HADES (Health Analytics Data to Evidence Suite) [6] for data analytics with data mapped to the OMOP CDM. In additions, there are also tools tailored for R users, such as Tidy R OMOP CDM ecosystem [7], which offers a tidyverse-like programming framework for conducting data analytics on OMOP-mapped data.

In this study, we aimed to develop an R package, named “CohortCharacteristics” [8], to facilitate cohort characterisation in datasets mapped to the OMOP CDM. The aim of CohortCharacteristics is to provide users with a suite of functions to generate tables and figures summarising cohort characteristics, such as demographics, comorbidities, medications, overlap between different cohorts, and timing of entries in the cohort.

To demonstrate the functionality and flexibility of this package, we conducted a characterisation study on new users of antipsychotic medications, dementia, and three different insomnia cohorts across seven European databases. The results were used for validation of the dementia and insomnia codelists definition of a clinical study requested by the European Medicines Agency as part of the DARWIN-EU initiative on the prescription trend [9] in of antipsychotic medications in dementia populations across Europe. DARWIN EU (Data Analysis and Real-World Interrogation Network) [10] is the European Medicines Agency’s federated network for conducting real-world evidence studies using data partners mapped to the OMOP CDM, enabling standardised analyses across multiple European healthcare databases.

Antipsychotic medications are widely prescribed but are often associated with significant safety concerns, particularly among vulnerable populations such as individuals with dementia or insomnia [11, 12]. Understanding the characteristics of these patient cohorts is therefore crucial, as it provides valuable insights that can guide clinical decision-making.

## Methods

### Package

#### Package details and software dependencies

CohortCharacteristics is an R package (R version 4.2.1) documented using “roxygen2”, and depends on the following existing packages: CDMConnector (≥ 1.6.0), “dplyr”, “tidyr”, “rlang”, “cli”, “stringr”, “omopgenerics” (≥ 0.4.0), visOmopResults (≥ 0.5.0), PatientProfiles (≥ 1.2.0), “snakecase”, “lifecycle”, and “purrr” [13–15]. CohortCharacteristics is designed for use with datasets mapped to the OMOP CDM, and therefore, datasets must be first converted to the OMOP CDM format before using the package. The package connects to various database management systems via the DBI R package.

To support a pipe-friendly syntax, CohortCharacteristics uses the omopgenerics [14] R package to manage CDM table references [16]. These references are stored within a single object, known as the CDM object, which is created from the DBI database connection and includes a list of references to the tables in the OMOP CDM. Full documentation on the CDM object and its interactions with the OMOP CDM can be found in the CDMConnector [16] and omopgenerics package documentation.

This R package was developed using a test-driven development approach [17]. Unit tests were created using a mock OMOP CDM object populated with synthetic test-specific data generated through internal helper function *mockCohortCharacteristics()* and with synthetic OMOP CDM tables from Eunomia dataset via the omock package [18, 19]. These unit tests included checks on the format and structure of output results and logical checks to ensure the accuracy of the analyses. Edge cases were also specifically tested with dedicated unit tests, including scenarios with missing data, empty cohort tables, and rare or inappropriate function input parameter combinations, to ensure robust handling of these situations. Informative error messages were added with the “cli” R package [20] to assist users when expected errors occurred. This ensures that CohortCharacteristics is both intuitive and user-friendly.

Development version with the documentation website can be found in the following GitHub repository: https://github.com/darwin-eu/CohortCharacteristics/.

#### Main functions

The central functions of the package are the six *summarise* functions:

1. summariseCohortCharacteristics: This function is used to summarise the cohort’s demographics and intersections with other cohorts or tables. The summarise demographics include: age (at user desired index date), age group, sex, prior observation (time in observation before cohort index date), future observation (time in observation after index date), duration (number of days in the cohort). The summarise intersections can be with cohorts, concept sets or tables. The intersection information can either be presence or not, number of counts, or time to event; a time window limits intersections. These are useful to calculate the percentage of subjects that took a certain medication and comorbidity in the year before the index date, the mean number of visits that the individuals had before entering the cohort or the meantime to a future event (e.g. outcome of interest). This is the primary function of the package.
2. summariseCohortCount: to extract the cohort counts metadata of a cohort.
3. summariseCohortAttrition: to extract the cohort attrition metadata of a cohort, cohort attrition mean the loss of individual for each step of the cohort creation.
4. summariseCohortOverlap: to identify common subjects (overlap) between cohorts.
5. summariseCohortTiming: to calculate the time between entries of different cohorts.
6. summariseLargeScaleCharacteristics: to extract any clinical events based on concept id recorded in the database, where a concept ID is the standard unique identifier used in the OMOP CDM to represent clinical terms such as conditions, drugs, or procedures, in certain time windows respecting the cohort index date. For example, to identify all the medications recorded in the month ([−30, −1]) before the index date.

The principal input of these functions is a cohort (patient level data, one row per entry), but the output contains summarised aggregated data. All functions provide the ability to stratify the results by columns of interest present in our cohort, e.g. sex or socioeconomic quantiles.

For each of the “summarise” functions described above, the associated “plot” and “table” functions allow users to visualise the results. These functions generate tables and graphs according to the type of the result. The names of the primary function, plot, and table functions are shown in Table 1.

**Table 1:** Main functions of the cohortcharacteristics package

Summarise functions	Plot functions	Table functions
summariseCharacteristics()	plotCharacteristics()	tableCharacteristics()
summariseCohortAttrition()	plotCohortAttrition()	tableCohortAttrition()
summariseCohortCount()	plotCohortCount()	tableCohortCount()
summariseCohortOverlap()	plotCohortOverlap()	tableCohortOverlap()
summariseCohortTiming()	plotCohortTiming()	tableCohortTiming()
summariseLargeScaleCharacteristics()	plotLargeScaleCharacteristics() plotComparedLargeScaleCharacteristics()	tableLargeScaleCharacteristics()

The package provides a dedicated vignette with code and examples to explain how to use each of the functions of the package [14].

### Clinical study

Using the package, we characterised five different cohorts across seven primary care and national health registry databases in Europe. The databases included in the study were IQVIA Longitudinal Patient Database Belgium (IQVIA LBD Belgium) [21], Integrated Primary Care Information Project (IPCI) Netherlands [22], The Information System for Research in Primary Care (SIDIAP) Spain [23], Danish Health Data Registries (DK-DHR) Denmark [24], IQVIA DA Germany [25], National Public Health Information System (NAJS) Croatia [26], and Clinical Practice Research Datalink GOLD (CPRD GOLD) United Kingdom [27]. All these databases were mapped to the OMOP CDM. Full descriptions of the databases included in the study can be found in the supplementary note 1.

We characterised 5 different cohorts:

- Individuals with dementia (dementia): individuals identified with dementia disease code.
- Individuals with insomnia (insomnia_broad): individuals with an insomnia code.
- Individuals taking antipsychotic medications (any_antipsychotics): Individuals are in the cohort while they have a prescription record of an antipsychotic medication.
- insomnia no dementia (insomnia_broad_no_prior_dementia): index date insomnia code, with no prior ([-Inf, −1/0]) record of dementia,
- insomnia dementia (insomnia_broad_prior_dementia): index date insomnia code, with prior ([-Inf, −1/0]) record of dementia,

To characterise these cohorts, we applied the summarise functions from CohortCharacteristics R package (described in Table 1) to generate a wide range of descriptive statistics. Specifically, we calculated cohort counts to quantify the number of individuals meeting each cohort definition. We applied the attrition function to document the application of inclusion and exclusion criteria at each step of the cohort-building process. The `summariseCohortCharacteristics` function was used to produce detailed “Table One” summaries, reporting key demographic variables such as age and sex. Also the prior observation time, comorbidity, and medication history before cohort entry. The comorbidity and medication cohorts codelist for “Table One” was pre-defined up-front by clinicians.

We also applied the package’s plotting and tabulation functions to support interpretation and facilitate cross-database comparisons. These functions generate standardised visualisations—such as bar plots of comorbidity prevalence, timing diagrams to illustrate the sequence of clinical events, and structured summary tables ready for reporting. Study code and code lists are available in the following Github repository: https://github.com/oxford-pharmacoepi/CohortCharacteristicsArticle.

## Results

### Package

CohortCharacteristics is freely available under the Apache License (Version 2.0) and can be obtained from CRAN (version 1.1.0 as of Nov 2025 https://cran.r-project.org/web/packages/CohortCharacteristics/index.html. The package offers detailed documentation, clear installation instructions, and practical vignettes to support users. As of April 2025, it has been downloaded over 14,000 times and achieves 95% unit test coverage. It has been particularly instrumental in studies commissioned by the European Medicines Agency through the DARWIN EU^®^ Coordination Centre [28–31] and is increasingly adopted in clinical research using OMOP CDM mapped data.

### Clinical study

All tables and figures shown for the clinical study in this section were created programmatically using the CohortCharacteristics package.

Table 2 presents the characteristics of individuals included in the dementia cohort across all seven databases. The cohort sizes vary across databases, but the mean age and sex distribution are consistent across all 7 databases.

**Table 2:** Demographic characteristics of individuals included in the dementia cohort across the seven European databases

		Database Name						
		CPRD GOLD	DK-DHR	IPCI	IQVIA Belgium LPD	IQVIA DA Germany	NAJS	SIDIAP
Number records	N	93,280	90,858	27,525	7,587	361,808	116,371	114,875
Number subjects	N	93,280	90,858	27,525	7,587	361,808	116,371	114,875
Cohort start date	Median [Q25–Q75]	2017-02-13 [2014-11-17–2020-01-16]	2018-05-03 [2015-09-05–2021-03-29]	2018-09-18 [2016-01-26–2021-04-19]	2016-10-12 [2014-09-25–2019-08-18]	2018-11-23 [2016-04-27–2021-03-18]	2018-12-28 [2016-07-30–2021-07-14]	2017-10-27 [2015-04-07–2020-09-10]
Age	Median [Q25–Q75]	83 [77–88]	81 [75–86]	82 [77–87]	81.00 [75.00–86.00]	82.00 [77.00–86.00]	81 [75–86]	83 [77–87]
	Mean (SD)	82.10 (8.18)	79.83 (8.82)	81.29 (8.36)	79.03 (10.87)	80.24 (9.72)	79.31 (9.89)	81.69 (8.27)
	Range	4 to 108	0 to 109	5 to 104	2 to 105	0 to 99	0 to 109	0 to 107
Sex (Female%)	N (%)	57,073 (61.18%)	52,316 (57.58%)	16,577 (60.23%)	4,721 (62.22%)	215,946 (59.69%)	77,368 (66.48%)	74,036 (64.45%)
Prior days of observation	Median [Q25–Q75]	4,683 [1,443–6,306]	8,459 [7,472–9,556]	1,376 [484–2,525]	274.00 [0.00–1,162.00]	56.00 [0.00–2,236.00]	1,487 [552–2,474]	4,156 [3,204–5,258]
	Mean (SD)	4,239.67 (2,826.03)	8,441.13 (1,437.64)	1,643.09 (1,334.19)	733.16 (957.33)	1,431.16 (2,254.80)	1,651.48 (1,404.87)	4,138.26 (1,395.29)
	Range	0 to 12,740	0 to 10,591	0 to 6,420	0.00 to 3,662.00	0.00 to 11,564.00	0 to 9,482	0 to 6,388
Future days of observation	Median [Q25–Q75]	553 [226–1,079]	909 [380–1,628]	579 [253–1,109]	891.00 [359.00–1,709.00]	368.00 [70.00–1,055.00]	595 [162–1,352]	1,017 [423–1,879]
	Mean (SD)	759.99 (703.40)	1,101.27 (886.42)	784.48 (706.17)	1,125.98 (912.34)	690.99 (814.91)	876.17 (871.99)	1,236.09 (960.10)
	Range	0 to 3,994	0 to 4,091	0 to 4,197	0.00 to 3,761.00	0.00 to 3,923.00	0 to 4,173	0 to 3,832
Number of visits in prior year	Median [Q25–Q75]	35 [22–52]	5 [2–10]	7 [3–14]	2 [0–7]	1 [0–7]	24 [10–41]	17 [9–29]
	Mean (SD)	38.78 (24.48)	7.69 (10.01)	9.85 (9.58)	4.56 (6.59)	5.61 (10.13)	28.78 (26.34)	21.44 (19.43)
	Range	0 to 321	0 to 263	0 to 114	0 to 66	0 to 162	0 to 571	0 to 493

The data include cohort sizes, mean age, and the proportion of female participants, providing insight into the population structure and comparability across databases

Table 3 reports the indications of common medication exposures within one year prior to first dementia diagnosis. CPRD GOLD (UK), NAJS (Croatia), and SIDIAP (Spain) consistently show higher percentages for major medication categories such as respiratory, cardiovascular, alimentary tract/metabolism, and nervous system medications, indicating similar prescribing patterns or underlying population characteristics in these databases. In contrast, the IQVIA databases (LPD Belgium and DA Germany) and DK-DHR (Denmark) generally report lower proportions in these categories, with particularly notable differences in the use of alimentary tract/metabolism and nervous system medications.

**Table 3:** This table summarises the prevalence of common medication exposures within one year before a first-time dementia diagnosis

	Database Name						
	CPRD GOLD	DK-DHR	IPCI	IQVIA Belgium LPD	IQVIA DA Germany	NAJS	SIDIAP
Antiparasitic products insecticides and repellents	4,318 (4.63%)	2,001 (2.20%)	238 (0.86%)	30 (0.40%)	2,225 (0.61%)	3,003 (2.58%)	1,278 (1.11%)
Respiratory system	29,302 (31.41%)	16,776 (18.46%)	6,859 (24.92%)	1,314 (17.32%)	35,859 (9.91%)	58,454 (50.23%)	42,100 (36.65%)
Sensory organs	16,976 (18.20%)	11,998 (13.21%)	3,935 (14.30%)	289 (3.81%)	5,447 (1.51%)	43,719 (37.57%)	46,526 (40.50%)
Blood and blood forming organs	45,555 (48.84%)	26,423 (29.08%)	9,851 (35.79%)	988 (13.02%)	39,464 (10.91%)	52,402 (45.03%)	49,712 (43.27%)
Antineoplastic and immunomodulating agents	3,819 (4.09%)	972 (1.07%)	942 (3.42%)	155 (2.04%)	3,292 (0.91%)	3,048 (2.62%)	5,573 (4.85%)
Alimentary tract and metabolism	66,494 (71.28%)	32,927 (36.24%)	18,558 (67.42%)	1,994 (26.28%)	75,823 (20.96%)	79,959 (68.71%)	86,457 (75.26%)
Musculo skeletal system	28,915 (31.00%)	14,610 (16.08%)	5,330 (19.36%)	1,088 (14.34%)	41,130 (11.37%)	43,799 (37.64%)	38,990 (33.94%)
Systemic hormonal preparations excl sex hormones and insulins	20,132 (21.58%)	8,690 (9.56%)	4,559 (16.56%)	606 (7.99%)	30,722 (8.49%)	17,203 (14.78%)	34,749 (30.25%)
Dermatologicals	33,044 (35.42%)	15,670 (17.25%)	7,835 (28.47%)	638 (8.41%)	27,891 (7.71%)	44,748 (38.45%)	30,975 (26.96%)
Various	20,546 (22.03%)	5,353 (5.89%)	2,331 (8.47%)	151 (1.99%)	6,247 (1.73%)	14,234 (12.23%)	7,049 (6.14%)
Cardiovascular system	70,346 (75.41%)	41,935 (46.15%)	19,280 (70.05%)	2,799 (36.89%)	98,488 (27.22%)	88,737 (76.25%)	91,468 (79.62%)
Nervous system	72,143 (77.34%)	41,864 (46.08%)	15,331 (55.70%)	3,007 (39.63%)	99,443 (27.49%)	75,158 (64.58%)	100,519 (87.50%)
Antiinfectives for systemic use	49,897 (53.49%)	40,530 (44.61%)	12,632 (45.89%)	1,538 (20.27%)	34,550 (9.55%)	63,909 (54.92%)	41,707 (36.31%)
Genito urinary system and sex hormones	22,873 (24.52%)	15,807 (17.40%)	5,107 (18.55%)	642 (8.46%)	14,786 (4.09%)	42,584 (36.59%)	25,610 (22.29%)

Table 4 presents the prevalence of common conditions at any time prior to dementia diagnosis. CPRD GOLD, DK-DHR, IPCI, NAJS, and SIDIAP report relatively high proportions, often exceeding 70% for major conditions such as circulatory and nervous system disorders. In contrast, the IQVIA databases consistently report lower prevalence for several condition groups, including neoplasms, infectious diseases, and symptoms not elsewhere classified.

**Table 4:** This table reports the prevalence of common pre-defined medical conditions anytime before a dementia diagnosis in each database

	Database Name						
	CPRD GOLD	DK-DHR	IPCI	IQVIA Belgium LPD	IQVIA DA Germany	NAJS	SIDIAP
Mental and behavioural disorders	23,485 (25.18%)	24,562 (27.03%)	4,833 (17.56%)	1,962 (25.86%)	88,495 (24.46%)	77,129 (66.28%)	57,710 (50.24%)
Diseases of the blood and blood forming organs and certain disorders involving the immune mechanism	43,475 (46.61%)	21,578 (23.75%)	5,136 (18.66%)	921 (12.14%)	53,607 (14.82%)	42,778 (36.76%)	41,312 (35.96%)
Neoplasms	29,817 (31.97%)	35,506 (39.08%)	8,730 (31.72%)	497 (6.55%)	50,330 (13.91%)	35,216 (30.26%)	40,575 (35.32%)
Diseases of the ear and mastoid process	28,843 (30.92%)	26,500 (29.17%)	10,683 (38.81%)	720 (9.49%)	38,329 (10.59%)	36,358 (31.24%)	54,384 (47.34%)
Diseases of the digestive system	48,773 (52.29%)	52,452 (57.73%)	11,603 (42.15%)	2,466 (32.50%)	92,063 (25.45%)	81,103 (69.69%)	80,078 (69.71%)
Endocrine nutritional and metabolic diseases	40,561 (43.48%)	43,626 (48.02%)	11,775 (42.78%)	2,883 (38.00%)	114,852 (31.74%)	66,325 (56.99%)	73,229 (63.75%)
Diseases of the nervous system	64,174 (68.80%)	73,134 (80.49%)	17,671 (64.20%)	3,312 (43.65%)	146,168 (40.40%)	92,072 (79.12%)	95,227 (82.90%)
Diseases of the musculoskeletal system and connective tissue	70,824 (75.93%)	80,101 (88.16%)	19,553 (71.04%)	3,190 (42.05%)	141,442 (39.09%)	96,653 (83.06%)	99,318 (86.46%)
Codes for special purposes	51,788 (55.52%)	4,538 (4.99%)	2,861 (10.39%)	1,828 (24.09%)	82,488 (22.80%)	48,311 (41.51%)	31,171 (27.13%)
Diseases of the eye and adnexa	46,163 (49.49%)	50,207 (55.26%)	12,081 (43.89%)	743 (9.79%)	52,051 (14.39%)	58,775 (50.51%)	68,166 (59.34%)
Certain infectious and parasitic diseases	76,596 (82.11%)	82,783 (91.11%)	23,105 (83.94%)	3,568 (47.03%)	151,598 (41.90%)	102,433 (88.02%)	106,555 (92.76%)
External causes of morbidity and mortality	33,957 (36.40%)	19,635 (21.61%)	1,395 (5.07%)	61 (0.80%)	25,576 (7.07%)	10,589 (9.10%)	6,941 (6.04%)
Diseases of the circulatory system	72,633 (77.87%)	68,044 (74.89%)	21,448 (77.92%)	3,860 (50.88%)	154,236 (42.63%)	103,041 (88.55%)	104,110 (90.63%)
Symptoms signs and abnormal clinical and laboratory findings not elsewhere classified	89,513 (95.96%)	88,977 (97.93%)	25,897 (94.09%)	4,731 (62.36%)	193,916 (53.60%)	109,061 (93.72%)	112,498 (97.93%)
Diseases of the skin and subcutaneous tissue	60,404 (64.76%)	43,823 (48.23%)	15,679 (56.96%)	1,804 (23.78%)	88,061 (24.34%)	73,327 (63.01%)	74,385 (64.75%)
Diseases of the genitourinary system	52,007 (55.75%)	49,868 (54.89%)	13,574 (49.32%)	1,986 (26.18%)	93,481 (25.84%)	79,091 (67.96%)	83,620 (72.79%)
Factors influencing health status and contact with health services	89,536 (95.99%)	86,838 (95.58%)	25,216 (91.61%)	4,507 (59.40%)	169,323 (46.80%)	104,698 (89.97%)	111,879 (97.39%)
Certain conditions originating in the perinatal period	30,947 (33.18%)	31,498 (34.67%)	8,603 (31.26%)	2,281 (30.06%)	96,599 (26.70%)	56,319 (48.40%)	54,179 (47.16%)
Diseases of the respiratory system	55,286 (59.27%)	53,220 (58.57%)	13,905 (50.52%)	2,842 (37.46%)	111,024 (30.69%)	81,414 (69.96%)	84,582 (73.63%)
Congenital malformations deformations and chromosomal abnormalities	4,402 (4.72%)	7,033 (7.74%)	691 (2.51%)	102 (1.34%)	29,456 (8.14%)	7,837 (6.73%)	10,238 (8.91%)
Injury poisoning and certain other consequences of external causes	77,162 (82.72%)	86,316 (95.00%)	22,669 (82.36%)	3,436 (45.29%)	154,657 (42.75%)	102,523 (88.10%)	106,761 (92.94%)

**Fig. 1:**
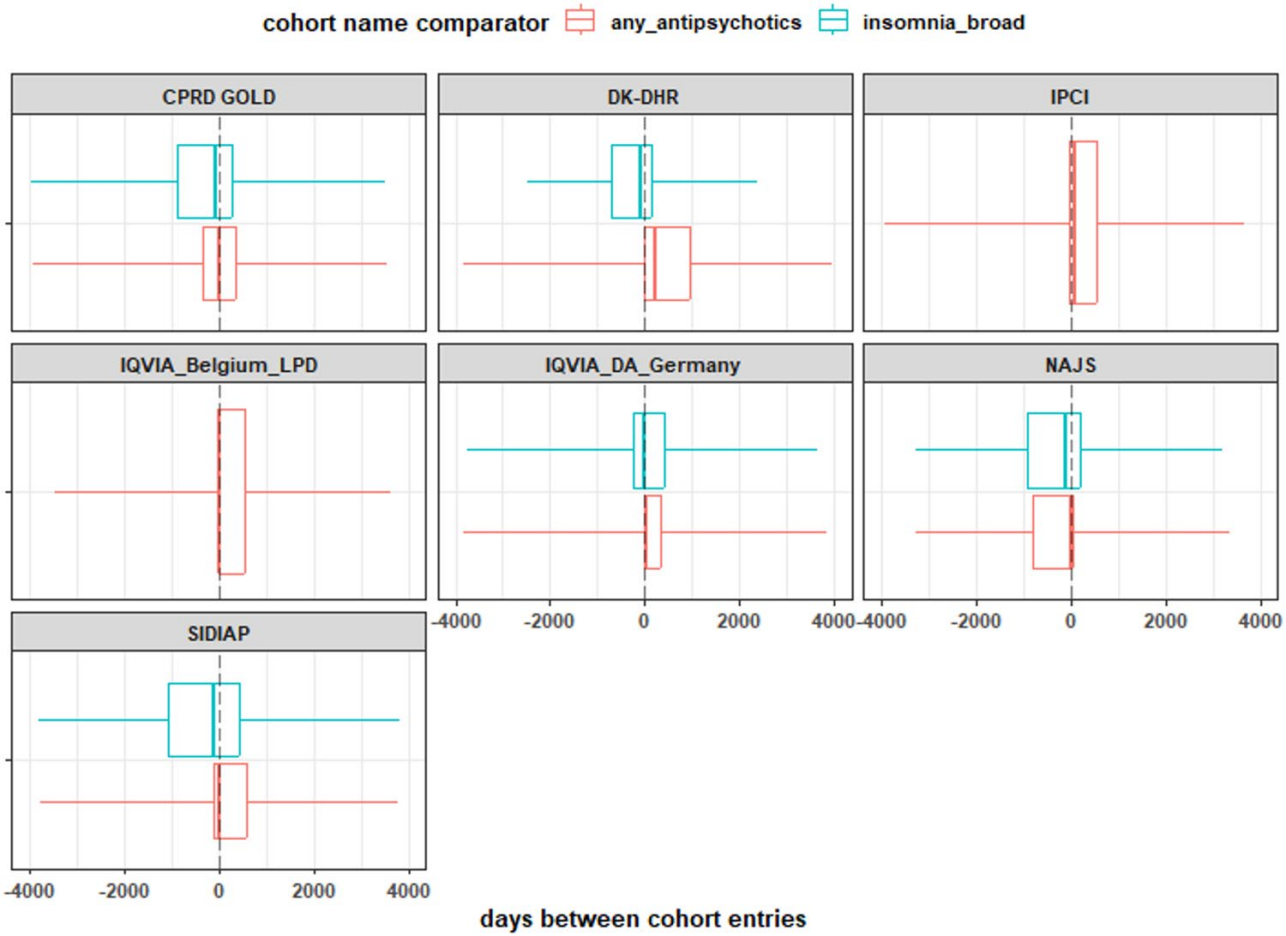
This figure shows the relative timing of cohort entry for individuals diagnosed with dementia, those prescribed antipsychotic medications, and those with insomnia across the seven databases

Figure 1 compares cohort entry dates for the dementia cohort against the antipsychotic and insomnia cohorts. In this figure, the boxplots show the distribution of index dates for the same individuals who appear in both the dementia and comparison cohorts (antipsychotic or insomnia). The position of each boxplot relative to the vertical black dotted line, which marks the dementia index date, indicates whether antipsychotic or insomnia cohort entry occurred earlier or later than the dementia diagnosis for the same individual. In CPRD GOLD, NAJS, DK-DHR, and SIDIAP, dementia diagnoses generally occur later than first-time insomnia diagnoses. Conversely, in DK-DHR, IPCI, IQVIA Belgium LPD, IQVIA Germany, and SIDIAP, antipsychotic medication use tends to follow dementia diagnosis.

**Fig. 2:**
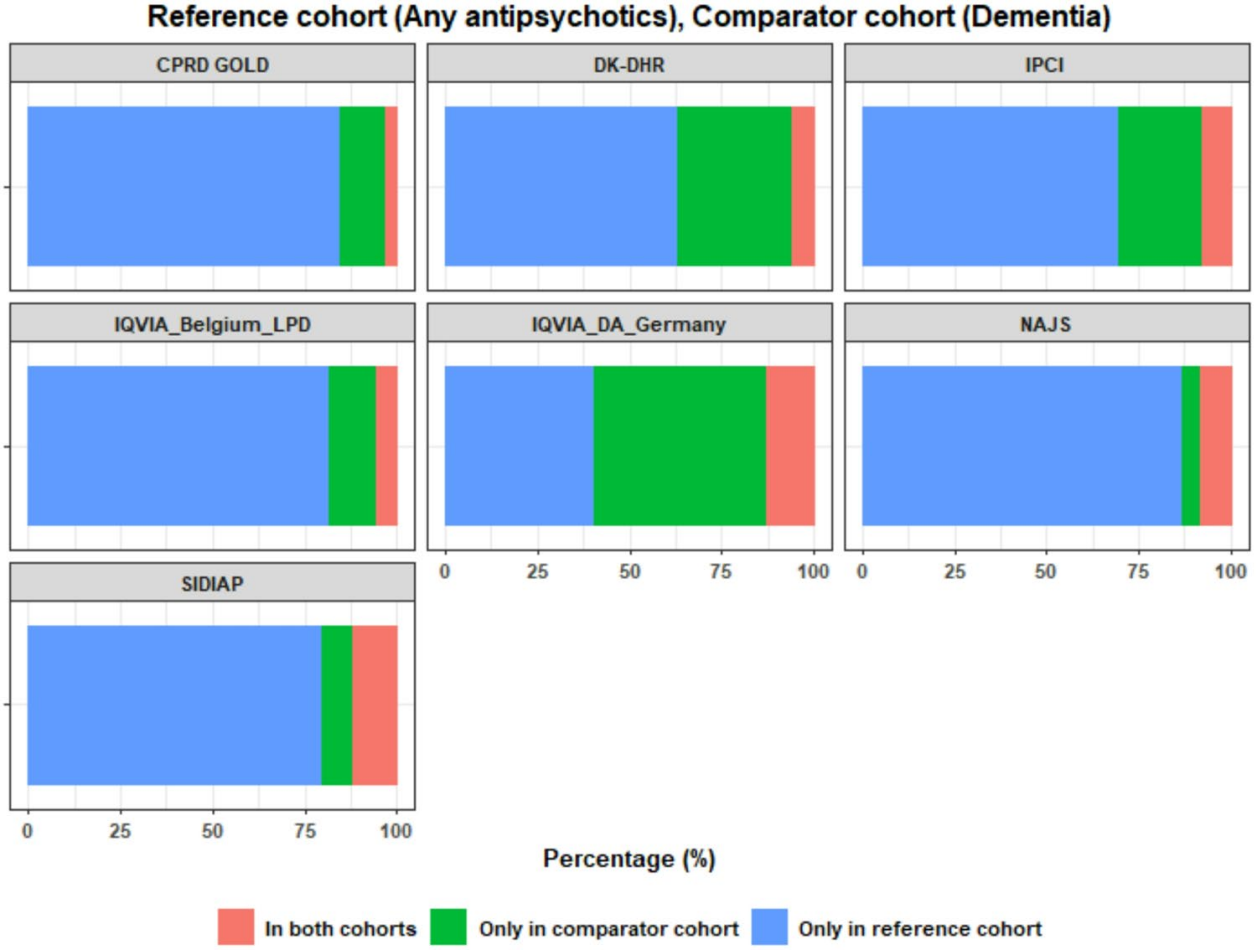
This figure shows the proportion of individuals overlapping in the dementia cohort and antipsychotic cohort

Figure 2 shows the cohort overlap plot between the dementia cohort and the antipsychotic medication user cohort, with the proportion of individuals in both cohorts ranging from 3.46% in CPRD GOLD to 13.57% in IQVIA DA Germany. Supplementary note 2 also shows the corresponding table for Fig. 2.

All results (including cohort attrition and database snapshots for each database and cohort analysed) can be visualised in more detail in a Shiny app: https://dpa-pde-oxford.shinyapps.io/cohortCharacteristicsArticle/.

## Discussion

### Summarise key findings

In this study, we developed and demonstrated the flexibility and functionality of CohortCharacteristics, an R package designed for cohort characterisation with datasets mapped to the OMOP CDM. The package’s flexibility is highlighted through its function arguments, allowing users to tailor analyses to their specific needs. For example, in the `summariseCohortCharacteristics()` function, users can specify a custom index date, define custom age groups, and set custom time windows for intersections with other cohorts or tables. The `summariseCohortOverlap()` and `summariseCohortTiming()` functions also allow users to summarise cohort overlap and the timing of entries between different cohorts. Furthermore, the `summariseCohortAttrition()` function can be used to provide information on attrition during cohort creation, and `summariseLargeScaleCharacteristics()` is helpful for summarising clinical events based on concept IDs recorded in the database.

To support reporting, the package also provides `plot` and `table functions for all summarise functions. These allow users to produce tables and figures suitable for publications and reports directly from the package outputs. These features collectively ensure that the package is suitable for cohort characterisation across a wide range of studies using OMOP CDM data.

By applying the package to characterise dementia, antipsychotic medication users, and insomnia cohorts across seven European databases, we illustrate its capability to generate comprehensive descriptive statistics, visualise cohort attributes, and its ability to facilitate cross-database comparisons for data mapped to the OMOP CDM.

The findings highlight variations in medication exposures and condition prevalence across databases for individuals with dementia. CPRD GOLD, NAJS, and SIDIAP consistently reported higher prescribing rates for respiratory, cardiovascular, alimentary tract/metabolism and nervous system medications, suggesting similar prescribing patterns or population characteristics. Conversely, IQVIA Belgium LPD, IQVIA DA Germany, and DK-DHR generally had lower proportions in these categories, particularly for alimentary tract/metabolism and nervous system medications. Similarly, we observed that the prevalence of common conditions before dementia diagnosis varied, with CPRD GOLD, DK-DHR, IPCI, NAJS, and SIDIAP showing higher rates of circulatory and nervous system disorders than the IQVIA databases.

The analysis of cohort entry timing suggests that in CPRD GOLD, NAJS, DK-DHR, and SIDIAP, first-time dementia diagnoses typically occurred later than insomnia diagnoses, supporting previous findings which suggested a potential link between sleep disturbances and dementia onset. This observation aligns with studies indicating that sleep disorders, such as insomnia, may elevate the risk of cognitive decline and dementia [32]. For instance, research has shown that individuals with sleep disorders have a 17% higher risk of developing dementia compared to those without sleep disturbances [33]. However, this pattern should not be interpreted as evidence of a causal relationship, as insomnia diagnoses commonly occur throughout adulthood, whereas dementia is typically diagnosed in older age [34, 35]. In all databases apart from NAJS, antipsychotic use followed dementia diagnosis. This pattern is consistent with NICE clinical guidelines, which recommend antipsychotics primarily for managing severe behavioural and psychological symptoms of dementia (BPSD) when non-pharmacological interventions are ineffective [36].

Furthermore, we identified a notable range in cohort overlap between dementia and antipsychotic medication users, with the proportion varying from 3.46% in CPRD GOLD to 13.57% in IQVIA DA Germany. This variation likely reflects differences in national prescribing practices, coding behaviour, and population characteristics across European healthcare systems. Higher overlap may indicate more frequent use of antipsychotics among people with dementia, whereas lower overlap may suggest more conservative prescribing patterns. However, these findings should be interpreted cautiously, as the primary purpose of this analysis is to support cohort definition diagnostics rather than to conclude prescribing behaviour without further analysis.

### Comparison with existing tools

ATLAS, a widely used OHDSI web-based platform [5], provides a user-friendly interface for defining cohorts and performing cohort characterisation. It also offers functionality for generating interactive descriptive summaries of cohorts through its graphical interface and is made for quick exploration and collaborative cohort development. However, ATLAS is designed primarily as a stand-alone software for users working through a graphical interface, and its outputs cannot easily be directly integrated into R programming code based analytical pipelines. In contrast, the CohortCharacteristics package enables cohort characterisation entirely within R, allowing integration with existing R programming code based workflows, for fully reproducible, scripted analyses. Furthermore, unlike ATLAS, which also supports cohort creations, CohortCharacteristics works with cohort tables that have already been created and populated in the OMOP CDM, typically using tools such as ATLAS (through exporting the cohort JSON file and instantiating with tools such as CDMConnector), CohortConstructor [37], or with bespoke R code. This allows the package to fit naturally into R-based workflows providing the user with flexibility and fitting naturally with other R packages [7, 14–16, 18, 37]. CohortCharacteristics should therefore be viewed as a complementary tool to ATLAS, providing an R programming-based, coding driven option for users who require performing their analysis in R.

### Strength and limitations

This study demonstrates the functionality of CohortCharacteristics, an open-source R package designed to standardise and automate cohort characterisation in OMOP CDM-mapped datasets. The package provides a structured and reproducible approach to summarising key cohort attributes, including demographics, comorbidities, medication exposures, cohort attrition, and temporal relationships between cohorts.

Despite its strengths, this package has some limitations. The main limitation is that CohortCharacteristics is only compatible with OMOP CDM-mapped data; hence, it cannot be applied to non-mapped health data. Also, while the package supports a wide range of cohort summary functions, it does not currently include built-in statistical testing such as standardised mean differences or t-tests. Future enhancements could expand its functionality to include more advanced analytical features.

### Interpretation and implications

This study underscores the value of analytic tools such as CohortCharacteristics for characterising cohorts in network studies using data mapped to the OMOP CDM. Demonstrating the package’s core functionality highlights its ability to streamline cohort characterisation, improve transparency, and support reproducibility in multi-database research. The ability to efficiently generate standardised cohort summaries across diverse datasets strengthens the reliability of cross-database comparisons and facilitates high-quality observational research.

The clinical results further support the validity of the cohort definitions for first-time dementia diagnoses and antipsychotic users, as the observed timing of cohort entries and medical histories align with established medical guidelines and existing literature.

## Conclusion

This study highlights the critical role of robust cohort characterisation in multi-database research and demonstrates the core functionality of CohortCharacteristics in facilitating such analyses. Variability in medication prescribing patterns and condition prevalence across databases underscores the importance of ensuring consistent and transparent cohort definitions when conducting observational studies. By providing a structured approach to summarising cohort attributes, CohortCharacteristics enables researchers to systematically assess cohort comparability, identify potential biases, and enhance the reliability of cross-database analysis.

## Supplementary Information

Below is the link to the electronic supplementary material.

Supplementary Material 1
