# Supplementary material for: CohortCharacteristics: an R package for population characterisation in observational studies using the OMOP common data model
Source: Eur J Epidemiol. 2026 Apr 3;41(4):507–17. doi: 10.1007/s10654-025-01352-4 (PMC13331811; doi:10.1007/s10654-025-01352-4)
Supplement: Supplementary file 1 — Supplementary Material 1 [file 10654_2025_1352_MOESM1_ESM.docx]

# Supplementary materials

**Supplementary Note 1: Description of the databases included in the study.**

1) The Information System for Research in Primary Care (SIDIAP) is a clinical database of anonymised patient records in Catalonia, Spain. The Spanish public healthcare system covers more than 98% of the population, and more than two thirds of the Catalan population see their GP at least once a year. The computerisation of the primary care patient records of the Catalan Health Institute (CHI) was complete in 2005. SIDIAP was designed to provide a valid and reliable database of information from clinical records of patients registered in primary care centres for use in biomedical research. SIDIAP contains data of anonymised patients’ healthcare records for nearly six million people (approximately 80% of the Catalan population) registered in 287 primary care practices throughout Catalonia since 2005. It includes data collected by health professionals during routine visits in primary care, including anthropometric measurements, clinical diagnoses (International Classification of Diseases 10th revision ICD-10), laboratory tests, prescribed and dispensed medications, hospital referrals, demographic and lifestyle information. It was previously shown that SIDIAP population is highly representative of the entire Catalan region in terms of geographic, age, and sex distributions. The high quality of these data has been previously documented, and SIDIAP has been successfully applied to epidemiological studies of key exposures and outcomes. Quality checks to identify duplicate patient IDs are performed centrally at each SIDIAP database update. Checks for logical values and data harmonisation are performed. For biochemistry data, consistency for measurements taken in different laboratories is assessed, and unit conversion is undertaken when needed.

2) Integrated Primary Care Information [IPCI] (Netherlands, Primary Care Database)

The Integrated Primary Care Information (IPCI) database is a longitudinal observational database containing routinely collected data from computer-based patient records of a selected group of GPs throughout the Netherlands (N=723). IPCI was started in 1992 by the department of Medical Informatics of the Erasmus University Medical Center in Rotterdam with the objective to enable better post marketing surveillance of medications. The current database includes patient records from 2006 on, when the size of the database started to increase significantly. In 2016, IPCI was certified as Regional Data Center. Since 2019 the data is also standardised to the Observational Medical Outcomes Partnership common data model (OMOP CDM), enabling collaborative research in a large network of databases within the Observational Health Data Sciences and Informatics (OHDSI) community. The primary goal of IPCI is to enable medical research. In addition, reports are generated to inform GPs and their organisations about the provided care. Contributing GPs are encouraged to use this information for their internal quality evaluation. The IPCI database is registered on the European Medicines Agency (EMA) ENCePP resources database (https://catalogues.ema.europa.eu/).

3) Danish Data Health Registries [DK-DHR] (Denmark, National Registry)

Danish health data is collected, stored and managed in national health registers at the Danish Health Data Authority and covers the entire population which makes it possible to study the development of diseases and their treatment over time. There are no gaps in terms of gender, age and geography in Danish health data due to mandatory reporting on all patients from cradle to grave, in all hospitals and medical clinics. Personal identification numbers enable linking of data across registers, so we have data on all Danes throughout their lives, regardless of whether they have moved around the country. High data quality due to standardisation, digitisation and documentation means that Danish health data is not based on interpretation. The Danish Health Data Authority is responsible for the national health registers and for maintaining and developing standards and classifications in the Danish healthcare system. Legislation ensures balance between personal data protection and use.

In the present data base, we have access to the following registries for the entire Danish population of 5.9 million persons from 1.1.1995: The central Person Registry, The National Patient Registry, The Register of Pharmaceutical Sales, The National Cancer Register, The Cause of Death registry, The Clinical Laboratory Information Register, COVID-19 test and vaccination Registries, The complete Vaccination registry. All data registered from 1.1.1995 will be included.

4) IQVIA Disease Analyzer Germany [IQVIA DA Germany] (Primary Care database)

Germany DA is collected from extracts of patient management software used by GPs and specialists practicing in ambulatory care settings. Data coverage includes 39.6 M cumulative person. Patient visiting more than one provider are not cross identified for data protection reasons and therefore recorded as separate in the system. Dates of service include from 1992 through present. Observation time is defined by the first and last consultation dates. Germany has no mandatory GP system and patient have free choice of specialist. Medications are recorded as prescriptions of marketed products. No registration or approval is required for medication utilisation studies.

5) IQVIA Longitudinal Patient Database Belgium [IQIVIA LPD Belgium] (Primary Care Database)

Belgium Longitudinal patient data (LPD) is collected from GP prescribing systems and contains patient records on all signs and symptoms, diagnoses and prescribed medications. The information recorded allows patients and doctors to be monitored longitudinally. Data are recorded directly in the LPD from doctors’ surgeries in real-time during patient consultations via a practice management software system. It is used in studies to provide various market insights such as treatment trends, patient pathway analysis and treatment compliance. The panel of contributing physicians (a stable 300 GPs) is maintained as a representative sample of the primary care physician population in Belgium according to three criteria known to influence prescribing: age, sex and geographical distribution. Currently, the database is covering 1.1 M cumulative patients and covers from 2012 through to the present. The panel consists of a stable 300 GPs that are geographically well spread. The total number of active GPs in Belgium is 15.602. The regional geographical spread of physicians in the LPD data is also representative of the distribution across the country: 57% GPs in the North (compared to 54% nationally), 31% in the South (33% nationally) and 12% in Brussels (13%). The provider of the data has more than 2.250 GPs under contract so in case of a drop out a replacement is easily found. Medications obtained over the counter by the patient outside the prescription system are not reported. No explicit registration or approval is necessary for medication utilisation studies.

6) Croatian National Public Health Information System [NAJS] (Croatia, Registry)

The National Public Health Information System (Croatian: Nacionalni javnozdravstveni informacijski sustav - NAJS) is an organised system of information services by Croatian Institute of Public Health. NAJS enables data collecting, processing, recording, managing and storing of health-related data from health care providers as well as production and management of health information. NAJS contains medical and public health data collected and stored in health registries and other health data collections including cancer registry, mortality, work injuries, occupational diseases, communicable and non-communicable diseases, health events, disabilities, psychosis and suicide, diabetes, medication abuse and others.

7) Clinical Practice Research Datalink GOLD [CPRD GOLD], United Kingdom (University of Oxford)

The Clinical Practice Research Datalink (CPRD-GOLD) is a governmental, not-for-profit research service jointly funded by the National Institute for Health and Care Research and the Medicines and Healthcare Products Regulatory Agency, a part of the Department of Health, United Kingdom (UK) (https://cprd.com). CPRD-GOLD comprises computerised records of all clinical and referral events in primary care in addition to comprehensive demographic information and medication prescription data in a sample of UK patients, with the most recent data being predominantly from Scotland (52% of practices) and Wales (28% of practices). The prescription records include information on the type of product, date of prescription, strength, dosage, quantity, and route of administration. Data from contributing practices are collected and processed into research databases. Quality checks on patient and practice levels are applied during the initial processing. Data are available for 20M patients, including 3.2M currently registered patients. Approval for this study was granted via the Research Data Governance Process.

**Supplementary Note 2:** Cohort overlap table shows the proportion of individuals overlap in the dementia cohort and antipsychotic cohort.

|  | | **Database name** | | | | | | |
| --- | --- | --- | --- | --- | --- | --- | --- | --- |
|  |  | CPRD GOLD | DK-DHR | IPCI | IQVIA Belgium LPD | IQVIA DA Germany | NAJS | SIDIAP |
|  |  | **Comparator cohort (any antipsychotic)** | | | | | | |
| **Reference cohort (dementia)** | Only in reference cohort | 72,046 (11.75%) | 75,522 (30.56%) | 20,058 (22.02%) | 5,130 (12.36%) | 279,165 (45.84%) | 39,312 (4.54%) | 44,812 (8.02%) |
|  | In both cohorts | 21,234 (3.46%) | 15,336 (6.21%) | 7,467 (8.20%) | 2,457  (5.92%) | 82,643 (13.57%) | 77,059 (8.90%) | 70,063 (12.54%) |
|  | Only in comparator cohort | 519,629 (84.78%) | 156,292 (63.24%) | 63,567 (69.78%) | 33,922 (81.72%) | 247,244 (40.59%) | 749,559 (86.56%) | 443,998 (79.45%) |
